# Incidence and trends of multiple myeloma (MM) in Brazil – 1988–2020

**DOI:** 10.3332/ecancer.2025.1997

**Published:** 2025-09-24

**Authors:** Dandara Menezes de Araujo Oliveira, Rossana Veronica Mendonza Lopez, Lady Paola Aristizabal Arboleda, Maria Paula Curado

**Affiliations:** 1 Epidemiology and Statistics Nucleus, International Research Center, A.C.Camargo Cancer Center, São Paulo 01509-010, Brazil; 2 Center for Translational Research in Oncology, Instituto do Câncer do Estado de São Paulo, Faculdade de Medicina da Universidade de São Paulo - FMUSP, São Paulo 01246-000, Brazil; 3 Research Epidemiologist, Epidemiology and Statistics Nucleus, International Research Center, A.C.Camargo Cancer Center, São Paulo 01509-010, Brazil

**Keywords:** multiple myeloma, incidence, epidemiology

## Abstract

**Background:**

Multiple myeloma (MM) is a chronic hematological malignancy caused by a differentiated plasma cell disorder (Pawlyn, 2019). As a consequence of population aging, there has been an increase in incidence rates (Turesson *et al* 2018). In 2022, there were 187,744 new cases (Bray *et al* 2024). The incidence of MM in Brazil has not been estimated by the National Cancer Institute (INCA).

**Objective:**

To analyse the incidence rates and trends of MM across states in Brazil from Population-Based Cancer Registries (PBCRs).

**Methodology:**

Information was extracted from PBCR/INCA for the 1988–2020 period. Sociodemographic data were extracted from records of the Brazilian Institute of Geography and Statistics. Age-standardised incidence rates were calculated using the Segi global standard population. Trend analysis was performed using Join point Regression, version 4.7.0.0.

**Results:**

The highest incidence rates of MM in males were observed in the cities of Natal (Rio Grande do Norte state) and Jaú (São Paulo state) at 3.55/100,000 and 2.9/100,000, respectively. In females, rates were highest in the cities of Natal (Rio Grande do Norte state) and Aracajú (Sergipe state) at 2.66/100,000 and 2.21/100,000, respectively. Trends showed an annual increase of 10.45% in Campinas for males and 9.04% for females. Median age at diagnosis in Brazil was 65 years for both sexes, while the North region had the lowest average age at 63.2 years, and the South region the highest at 68.0 years. Porto Alegre city (Rio Grande do Sul state) had the highest average of 70.0 years for females and 67.1 for males, while Roraima had the lowest at 61.2 years for females and 54.2 for males.

**Conclusion:**

In Brazil, the average age of incidence varies by geographic region, but is higher among males. Incidence rates are highest in the Northeast and Southeast regions, whereas the greatest upward trends are in the Southeast and Midwest regions.

## Introduction

Multiple myeloma (MM) is a chronic hematologic malignancy caused by a differentiated plasma cell disorder [1]. The condition is characterised by renal failure, hypercalcemia, lytic bone lesions and anemia [2]. Risk factors for MM include family history, exposure to radiation or chemical products, monoclonal gammopathy of undetermined significance, obesity, male gender, age >60 years and African ancestry [3–5].

The disease, although treatable, has no established cure [6]. Treatment of the myeloma can take the form of high-dose combination chemotherapy (melphalan) with different therapeutic class medications: proteasome inhibitors, immunomodulators (thalidomide and lenalidomide) and monoclonal antibodies (daratumumab and elotuzumab) [7, 8].

In 2022, there were an estimated 187,744 new cases globally and 121,252 MM-related deaths [9]. The incidence of MM is 1.5 times higher in males and twice as prevalent in black individuals [10, 11]. Mean age at diagnosis is 70–75 years in Germany and the USA [11]. The age-standardised incidence rate of MM stood at 1.78/100,000 people in 2022 and the highest incidence has been reported in Australia and New Zealand (4.86), North America (4.74) and Northern Europe (3.82) [12].

In countries such as Norway, where the proportion of older adults in the population is higher than in the rest of the world, MM incidence rose from 7.3/100,000 in 1999 to 8.4/100,000 in 2017, compared with an increase from 3.6/100,000 to 4.2/100,000 among the world population [13]. In the USA, MM is the second-most-common hematological neoplasm, with 13,000 new cases in 2022, a figure projected to reach 32,000 by 2032–2034, driven by population aging [9, 14].

In South America, the disease had an estimated incidence of 2.0/100,000 population and mortality of 1.5/100,000 in both genders [15]. For Brazil, the estimated incidence in 2022 was 1.9/100,000, projected to reach 5.6/100,000 by 2045 [9]. Brazil is geographically divided into five regions (North, Northeast, Midwest, Southeast and South), with distinct demographic, socioeconomic and health access profiles. The Southeast and Northeast concentrate the largest Black and mixed-race populations, which is relevant given the higher MM risk in these groups [16].

Myeloma is a rare neoplasm that predominantly affects older adults. Brazil has a miscegenated population of whites and blacks and a life expectancy of 76 years. MM is a rare cancer and investigating the incidence of this neoplasm in Brazil can help identify regional patterns and at-risk populations. This study aimed to describe the incidence and analyse time trends of MM in Brazil.

## Methods

A descriptive, ecological, time-series study of data from the Brazilian Population-Based Cancer Registry (PBCR) was carried out. The cases were identified on the National Cancer Institute database (https://www.gov.br/inca/pt-br/assuntos/cancer/numeros/registros/base-populacional). Demographic data used for calculating incidence were extracted from the Brazilian Institute of Geography and Statistics (IBGE) [16].

All cases of MM registered on the PBCR between 1988 and 2019 were included. Topography was selected using the ICD-10 as C90. Cases with missing data on age, sex or geographical location were excluded.

Crude incidence rates were calculated by dividing the number of new cases of MM by the total population, as per data from the IBGE. Age-standardised incidence rates were calculated using the Segi world standard population, modified by Doll. Rates were expressed per 100,000 population annually and stratified by regions of Brazil, allowing analysis of regional variations in MM incidence nationwide.

Incidence trends were estimated by calculating the Average Annual Percent Change (AAPC). AAPC is a weighted average of the slope coefficients of the line regression model, with weights equal to the length of each interval segment. An increase or decrease in a trend is statistically significant when the 95% confidence interval (95% CI) does not include the null value (zero). Analysis of tendencies was performed using the Joinpoint Regression program, version 4.7.0.0 [17].

## Results

**Figure 1.**
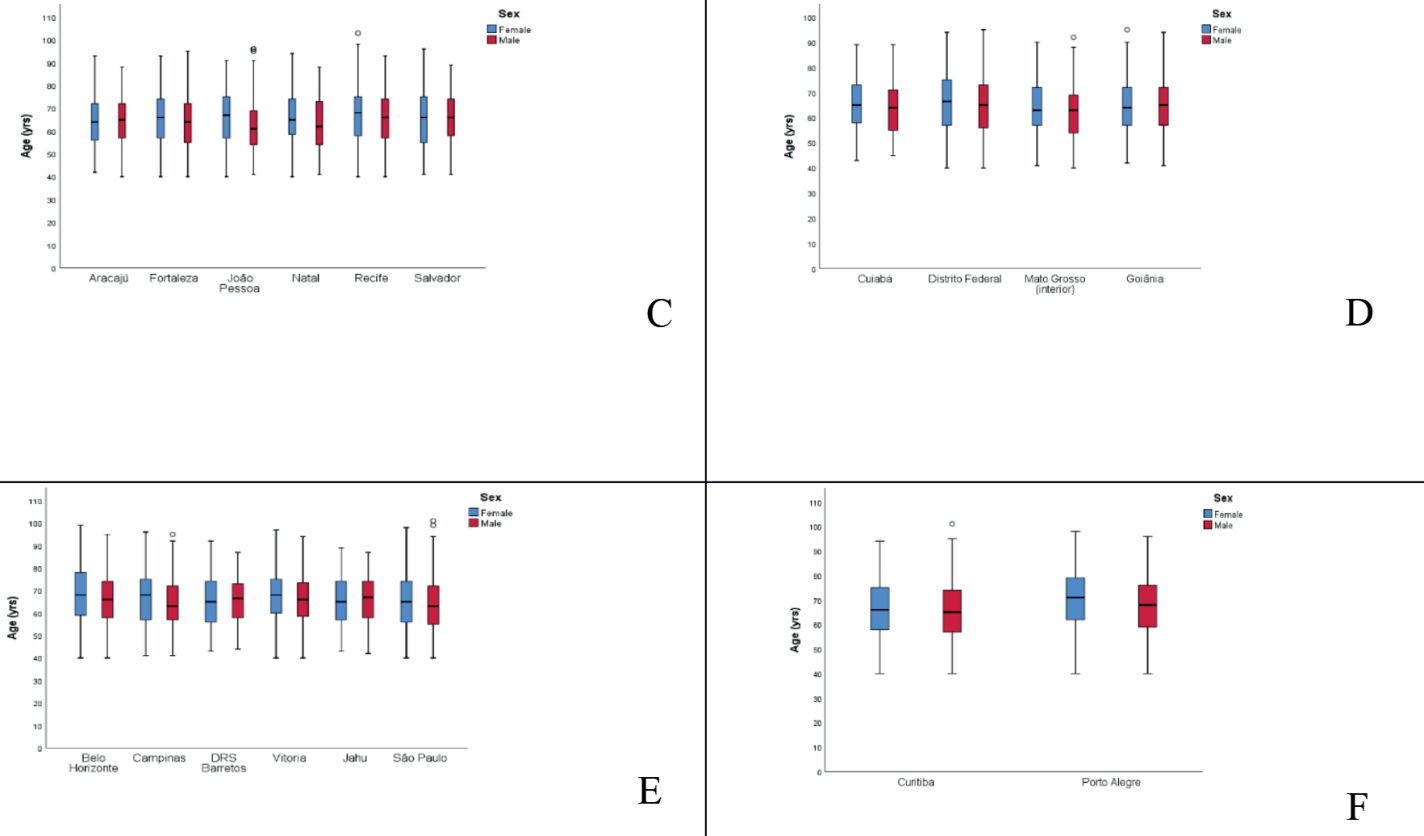
(a): Median age recorded of MM cases in Brazil by sex. (b): Median age recorded of MM cases in North by sex. (c): Median age recorded of MM cases in Northwest by sex. (d): Median age recorded of MM cases in Midwest by sex. (e): Median age recorded of MM cases in Southwest by sex. (f): Median age recorded of MM cases in South by sex.

The mean age of MM patients in Brazil was 61–70 years for both genders. Regionally, the North had the lowest mean age at diagnosis (63.2 years) and the South the highest (68.0 years). Porto Alegre (RS) had the highest mean age (70.0 years in women; 67.1 in men) and Roraima the lowest (61.2 women; 54.2 men) (Figure 1/Supplementary Table 1).

**Figure 2.**
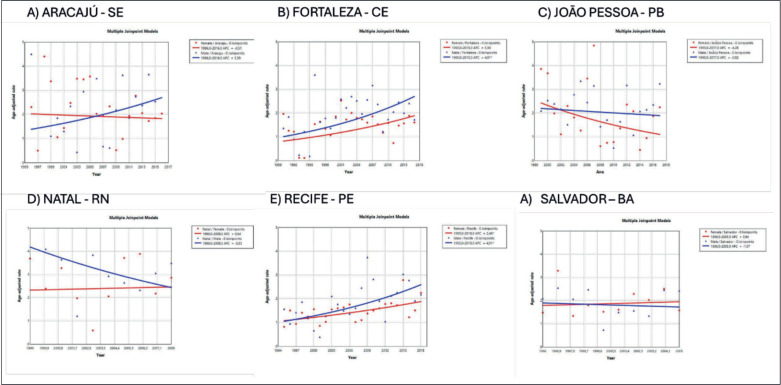
AAPC in the incidence of MM by sex in state capitals of the Northeast region of Brazil, 1988–2019. (a): Aracaju-SE. (b): Fortaleza-CE. (c): João Pessoa-PB. (d): Natal-RN. (e): Recife-PE. (f): Salvador-BA.

**Figure 3.**
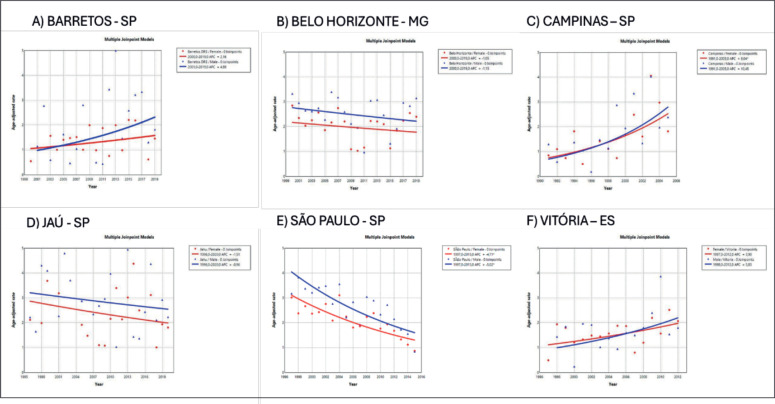
AAPC in the incidence of MM by sex in cities of the Southwest region of Brazil, 1988–2019. (a): Barretos – SP. (b): Belo Horizonte – MG (c): Campinas – SP. (d): Jaú – SP. (e): São Paulo – SP. (f): Vitória – ES.

In Brazil, the highest incidence rates of MM in males were observed in the cities of Natal (Rio Grande do Norte state) at 3.55/100,000 (Figure 2) and Jaú (São Paulo state) at 2.9/100,000 (Figure 3). The highest rates in females were in Natal (Rio Grande do Norte state) at 2.66/100,000 (Figure 2) and Aracajú (Sergipe state) at 2.21/100,000 (Figure 2).

**Figure 4.**
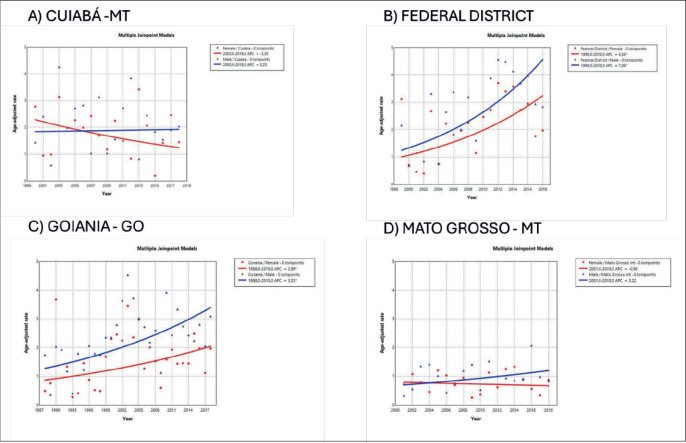
AAPC in the incidence of MM by sex in state capitals of the Midwest region of Brazil, 1988–2019. (a): Cuiabá – MT (b): Federal District – DF. (c): Goiânia – GO. (d): Mato Grosso – MT.

**Figure 5.**
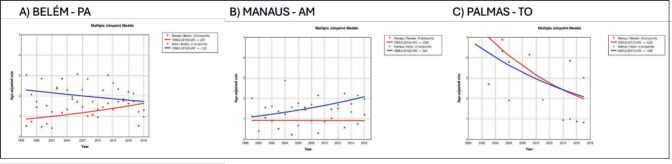
AAPC in the incidence of MM by sex in state capitals of the North region of Brazil, 1988–2019. (a): Belém – PA. (b): Manaus – AM. (c): Palmas – TO.

**Figure 6.**
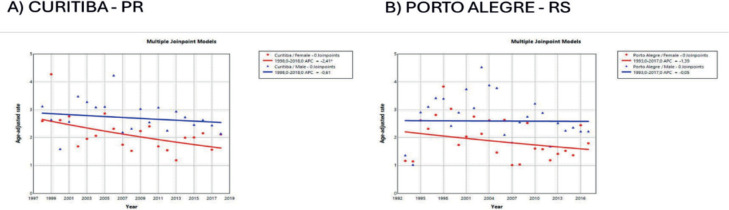
AAPC in the incidence of MM by sex in state capitals of the South region of Brazil, 1988–2019. (a): Curitiba – PR. (b): Porto Alegre – RS.

The trend in incidence, as measured by AAPC, was highest in the Federal District at 7.06% (95%CI: 1.80; 12.62) in males and 6.36% (95%CI: 1.48; 11.50) in females (Figure 4). There was a rising trend in the Northeast for males of 4.01%, but a decline in the Southeast (Table1, Figures 2–6).

## Discussion

The incidence and trend of MM in Brazil have increased heterogeneously across regions. The highest rise in incidence has occurred in the Midwest and Northeast, while the highest rates can be seen in the South and Southeast of the country. There is a predominance of cases in males (2.39/100,000 versus 1.88/100,000 in women) nationwide although ethnic miscegenation differs across regions.

These results corroborate the findings of Smith *et al* [10] and Turesson *et al* [14], showing that the population profile of MM is predominantly male. However, a study conducted in French Guyana over 2005–2014 revealed a different pattern, with a higher incidence found among women than men. This pattern might be explained by the high African ancestry of the population and growing rate of obesity, particularly among women [5].

In Brazil, the mean age at diagnosis and incidence of MM were lowest in the North region and highest in the South. In 2010, life expectancy was 70.8 years in the North and 75.5 years in the South, averaging 73.48 years in Brazil as a whole. These differences might reflect disparities in living conditions, such as access to healthcare and other socioeconomic factors influencing longevity across regions of Brazil [16].

MM is a disease most affecting the old, with 70% of cases in individuals aged 65 years or over [18]. The mean age at diagnosis in Brazil was 68 years (Median – 67.5) in the South and 63.2 years (Median – 61) in the North. In Colombia, the mean age at diagnosis was 67 years [19]. In more developed countries, such as Germany and the United Kingdom, the mean age of incidence is over 70 years [9, 20] whereas in Norway the mean age in 71 years [13]. Mean age at diagnosis can vary according to sex where, in studies by Joshi *et al* [8] performed in the USA over 2010–2014, median age was 63.0 years and 56.7% of cases were male.

A population-based study in India also found the highest incidence in the 60–69 years age group, as well as regional and gender differences, with an incidence in males of 1.13 (95% CI: 1.07–1.20) and females of 0.81 (95% CI: 0.75–0.88) per 100,000 [21]. In the present study, the highest incidence rates of MM in Brazil occurred in the Northeast region. These rates are 3 times lower than those recorded in the USA of 15.9 in black individuals and 7.5 in whites [22].

A study carried out in the USA over a 21-year period (1999–2020) found a steady increase in the incidence of MM, with substantial racial and ethnic disparities [23]. Non-Hispanic Black individuals exhibited the highest incidence rates, rising from 12.02/100,000 population in 1999 to 14.20/100,000 in 2020. By comparison, other ethnicities such as Non-Hispanic American Indian/Native Alaskans and Asian/Pacific Islanders had the lowest incidence rates of 5.59 and 3.56/100,000 in 1999, increasing to 5.76 and 3.92/100,000, respectively, by 2020 [23].

According to Curado *et al* [24], this heterogeneous pattern of incidence of MM might be related to limited access to diagnosis and treatment and gaps in the PBCR. In the present analysis, patients were not stratified by race because the database precluded this breakdown, representing a limitation of the study. According to the Instituto Brasileiro de Geografia e Estatística [29], Brazil has a racial composition of 47.73% white, 43.13% brown and 7.61% black individuals. In the North, the composition is 72.3% brown, 19.5% white and 7% black, while the Southeast constitutes 52.2% white, 37.6% brown and 9% black.

Besides ethnic differences, disparities by gender, age and region were also evident, underscoring the importance of targeted interventions and MM screening initiatives for at-risk populations [23]. In the present study, MM incidence was higher in males, with men accounting for 54.6% of cases (2.39 versus 1.88 in women). Similarly, in China, rates between 2012 and 2016 were higher in men than in women (1.84 versus 1.30) [25]. In Spain over a 23-year period, 51% of MM patients were male and 57.9% aged over 70 years [26].

The literature shows a rising trend in MM incidence in males, individuals aged ≥50 years and among high-income countries, such as Germany (AAPC 6.71 – 95% CI: 0.75–13.02), Denmark (3.93-CI 2.44–5.45) and South Korea (3.25 – CI 0.69–5.88) [12]. In Norway, MM incidence rose from 7.3 in 1999 to 8.4 in 2017, whereas incidence rate increased from 3.6 to 4.2 [13]. The results of the present investigation revealed an upward trend in incidence in the Northeast, Federal District and the Southeast. This rising trend in incidence observed in some regions of Brazil, such as the Southeast and Midwest, might be associated with greater access to healthcare and diagnosis or higher educational level, as reported in the study by Galvis *et al* [27].

In the present study, disparities in trends of increase in MM by region and gender were found. Trends of increase were found to be higher in males from cities such as Fortaleza (Ceara state), Recife (Pernambuco state), Goiânia (Goias state) and Campinas (São Paulo state). The study of Imounga *et al* [5] conducted in French Guyana in 2005–2014, showed a singular pattern with a trend of increasing incidence over time among women (from 5.88 to 9.5 cases per 100,000), exceeding the rise recorded in men (from 5.18 to 6.7 cases per 100,000 individuals), a phenomenon possibly associated with high prevalence of African ancestry and increasing rates of obesity among women in the country [5]. This highly increasing rate was also seen in the USA over 2000–2019 in women aged under 55 years, a group exhibiting a greater increase compared to men of the same age [22]. In Brazil in 2010, obesity rates more than tripled among women aged 18–24 and 55–64 years, declining only slightly among those aged ≥65 years [28].

## Conclusion

In Brazil, the mean age-adjusted incidence varied by geographic region and was higher in men. Highest incidence rates were found in the Northeast and Southeast regions, whereas the highest trends of increase occurred in the Southeast and Midwest. This study has some limitations related to the data from the population-based registries, such as under-notification and differences in data quality across regions.

Based on these findings, we recommend expanding cancer registration coverage and quality, especially in less-represented regions, as well as improving early diagnosis policies and public knowledge of MM. Public health strategies should consider regional and demographic disparities to improve equity in cancer surveillance and care.

## Conflicts of interest

The authors declare that they have no known competing financial interests or personal relationships that could have appeared to influence the work reported in this article.

## Author contributions

**Dandara Menezes de Araujo Oliveira:** Conceptualisation; Formal analysis; Methodology; Visualisation; Writing - original draft. **Rossana Veronica Mendonza Lopez:** Data curation; Investigation. **Lady Paola Arboleda:** Software. **Maria Paula Curado:** Supervision; Project administration; Validation; Writing - review & editing.

## Figures and Tables

**Table 1.** Age-standardised incidence rates for MM and AAPC by sex and state Brazilian PBCR (1988–2020).

City PBCR (state)	Period	Male			Female		
		Incidence	AAPC (95%CI)	p-value	Incidence	AAPC (95%CI)	*p*-value
North							
Manaus (AM)	1999–2016	1.64	3.84 (−0.40; 8.26)	0.082	1.01	−0.06 (−6.62; 7.08)	0.98
Belém (PA)	1996–2019	2.10	−1.22 (−3.93; 1.50)	0.37	1.34	2.81 (−0.57; 6.26)	0.10
Palmas (TO)	2001–2017	2.23	−4.90 (−14.91; 8.15)	0.4	1.96	−6.38 (−18.01; 7.81)	0.33
Northeast							
Salvador (BA)	1996–2005	1.92	−1.07 (−11.71; 11.09)	0.85	1.94	0.94 (−7.21; 9.61)	0.82
Fortaleza (CE)	1990–2015	1.92	4.01 (0.11; 8.03)*	**0.04**	1.42	3.38 (−1.23; 8.30)	0.16
João Pessoa (PB)	1999–2017	2.21	−0.82 (−5.35; 3.82)	0.72	1.99	−4.28 (−12.08; 4.11)	0.3
Recife (PE)	1995–2018	1.83	4.01 (1.02; 7.06)*	**0.009**	1.49	2.46 (0.60; 4.33)	0.009
Natal (RN)	1999–2016	3.55	−5.83 (−17.11; 6.95)	0.34	2.66	0.64 (−14.64; 18.98)	0.94
Aracajú (SE)	1996–2016	2.17	3.39 (−2.63; 9.93)	0.29	2.21	−0.51 (−5.07; 4.08)	0.82
Mid-west							
Goiânia (GO)	1988–2018	2.34	3.32 (1.18; 5.54)*	**0.002**	1.57	2.89 (0.04; 5.88)*	**0.05**
Cuiabá (MT)	2000–2018	2.12	0.25 (−4.81; 4.60)	0.91	2.05	−3.35 (−10.03; 3.73)	0.33
Mato Grosso int (MT)	2001–2018	1.01	3.22 (−3.47; 10.40)	0.36	0.81	−0.96 (−5.72; 4.30)	0.7
Federal District	1999–2018	2.75	7.06 (1.80; 12.62)*	**0.01**	2.14	6.36 (1.48; 11.50)*	**0.01**
Southeast							
Vitória (ES)	1997–2012	1.58	5.83 (−2.70; 15.54)	0.21	1.59	3.90 (−0.58; 8.52)	0.09
Belo Horizonte (MG)	2000–2019	2.58	−1.15 (−4.08; 1.90)	0.45	2.05	−1.05 (−3.61; 1.59)	0.42
Barretos DRS (SP)	2000–2019	1.68	4.88 (−3.84; 15.03)	0.31	1.29	2.16 (−1.83; 6.50)	0.3
Campinas (SP)	1991–2005	1.73	10.45 (0.12; 22.28)	0.06	1.53	9.04 (2.34; 16.36)*	**0.01**
Jaú (SP)	1996–2020	2.90	−0.96 (−4.38; 2.64)	0.59	2.12	−1.51 (−5.55; 2.83)	0.48
São Paulo (SP)	1997–2015	2.69	−**5.02 (**−**7.20;** −2.80)*	**< 0.01**	2.11	−**4.71 (**−**6.42;** −2.95)*	**< 0.01**
South							
Curitiba (PR)	1998–2018	2.77	−0.61 (−2.37; 1.15)	0.49	2.16	−**2.41 (**−4.53; -0.29)*	**0.02**
Porto Alegre (RS)	1993–2017	2.74	−0.05 (−2.13; 2.10)	0.96	2.00	−1.39 (−3.86; 1.22)	0.28

* *p* < 0.05

Bold values indicate statistically significant differences (*p* < 0.05)

## Supplementary table

**Supplementary Table 1.** Mean and median age by sex and record of MM cases in Brazil.

Region	City PBCR (state)	Female	Male	*p*-value
		Mean (median)	Mean (median)	
North	Belém	66.9 (67.0)	64.8 (65.0)	0.008*
	Manaus	62.4 (63.0)	62.5 (63.0)	0.71
	Palmas	62.3 (59.0)	60.1 (60.5)	0.56
	Roraima	61.2 (57.0)	54.2 (54.0)	0.18
Northeast	Aracajú	64.1 (64.0)	65.3 (65.0)	0.34
	Fortaleza	65.7 (66.0)	63.8 (64.0)	0.04*
	João Pessoa	66.3 (67.0)	62.7 (61.0)	0.03*
	Natal	67.0 (65.0)	63.0 (62.0)	0.09
	Recife	67.2 (68.0)	65.8 (66.0)	0.02*
	Salvador	65.2 (66.0)	65.7 (66.0)	0.76
Center-West	Cuiabá	65.1 (65.0)	63.1 (64.0)	0.11
	Distrito Federal	66.1 (66.5)	64.8 (65.0)	0.06
	Mato Grosso (interior)	64.3 (63.0)	62.4 (63.0)	0.31
	Goiânia	64.3 (64.0)	64.9 (65.0)	0.66
Southeast	Belo Horizonte	68.3 (68.0)	66.1 (66.0)	<0.01*
	Campinas	66.9 (68.0)	65.2 (63.0)	0.71
	DRS Barretos	65.0 (65.0)	65.8 (66.5)	0.89
	Espírito Santo	68.3 (68.0)	66.0 (66.0)	0.09
	Jahu	65.6 (65.0)	65.1 (67.0)	0.97
	São Paulo	64.8 (65.0)	63.5 (63.0)	<0.01*
South	Curitiba	66.1 (66.0)	65.1 (65.0)	0.15
	Porto Alegre	70.0 (71.0)	67.1 (68.0)	<0.01*

* <0.05

Bold values indicate statistically significant differences (*p* < 0.05)

## References

[1] Pawlyn C, Davies FE (2019). Toward personalized treatment in multiple myeloma based on molecular characteristics. Blood.

[2] Cowan AJ (2018). Global burden of multiple myeloma: a systematic analysis for the Global Burden of Disease Study. JAMA Oncol.

[3] Du Z, Weinhold N, Song GC (2020). A meta‐analysis of genome‐wide association studies of multiple myeloma among men and women of African ancestry. Blood Adv.

[4] Visram A, Cook J, Warsame R (2021). Smoldering multiple myeloma: evolving diagnostic criteria and treatment strategies. Hematol Am Soc Hematol Educ Program.

[5] Imounga LM, Drak Alsibai K, Plenet J (2023). The singular epidemiology of plasmacytoma and multiple myeloma in French Guiana. Cancers.

[6] Rajkumar SV (2022). Multiple myeloma: 2022 update on diagnosis, risk stratification, and management. Am J Hematol.

[7] Palumbo A (2015). Revised international staging system for multiple myeloma: a report from International Myeloma Working Group. J Clin Oncol.

[8] Joshi H, Lin S, Fei K (2021). Multiple myeloma, race, insurance and treatment. Cancer Epidemiol.

[9] Bray F, Laversanne M, Sung H (2024). Global cancer statistics 2022: GLOBOCAN estimates of incidence and mortality worldwide for 36 cancers in 185 countries. CA Cancer J Clin.

[10] Smith CJ, Ambs S, Landgren O (2018). Biological determinants of health disparities in multiple myeloma. Blood Cancer J.

[11] Pulte D, Jansen L, Castro FA (2015). Trends in survival of multiple myeloma patients in Germany and the United States in the first decade of the 21st century. Br J Haematol.

[12] Huang J, Chan SC, Lok V (2022). The epidemiological landscape of multiple myeloma: a global cancer registry estimate of disease burden, risk factors, and temporal trends. Lancet Haematol.

[13] Langseth ØO, Myklebust TÅ, Johannesen TB (2020). Incidence and survival of multiple myeloma: a population based study of 10,524 patients diagnosed 1982–2017. Br J Haematol.

[14] Turesson I, Bjorkholm M, Blimark CH (2018). Rapidly changing myeloma epidemiology in the general population: increased incidence, older patients, and longer survival. Eur J Haematol.

[15] Bull FC, AlAnsari SS, Biddle S (2020). World Health Organization 2020 guidelines on physical activity and sedentary behaviour. Br J Sports Med.

[16] Datasus, Departamento de Informática do SUS (2010). Informação em Saúde: Demográfica e Socioeconômica.

[17] Kim HJ, Fay MP, Feuer EJ (2000). Permutation tests for join point regression with applications to cancer rates. Stat Med.

[18] Jones A, Bowcock S, Rachet B (2021). Survival trends in elderly myeloma patients. Eur J Haematol.

[19] Abello V (2022). Realworld evidence of epidemiology and clinical outcomes in multiple myeloma, findings from the registry of Hematooncologic malignancies in Colombia, observational study. Clin Lymphoma Myeloma Leuk.

[20] Mohty M, Knauf W, Romanus D (2020). Real world treatment patterns and outcomes in nontransplant newly diagnosed multiple myeloma in France, Germany, Italy, and the United Kingdom. Eur J Haematol.

[21] Bora K (2019). Distribution of multiple myeloma in India: heterogeneity in incidence across age, sex and geography. Cancer Epidemiol.

[22] Mousavi SE, Ilaghi M, Aslani A (2023). A population based study on incidence trends of myeloma in the United States over. Sci Rep.

[23] Zhu DT, Park A, Lai A (2024). Multiple myeloma incidence and mortality trends in the United States, 1999–2020. Sci Rep.

[24] Curado MP, Oliveira MM, Silva DRM (2018). Epidemiology of multiple myeloma in 17 Latin American countries: an update. Cancer Med.

[25] Wang S, Xu L, Feng J (2020). Prevalence and incidence of multiple myeloma in urban area in China: a national population based analysis. Front Oncol.

[26] ChangChan DY, RíosTamayo R, Rodríguez Barranco M (2021). Trends of incidence, mortality and survival of multiple myeloma in Spain. A twenty three year population based study. Clin Transl Oncol.

[27] Galvis M, Tjioe KC, Balas EA (2023). Disparities in survival of hematologic malignancies in the context of social determinants of health: a systematic review. Blood Adv.

[28] Ministério da Saúde and Secretaria de Vigilância em Saúde (2010). VIGITEL 2010: Vigilância de Fatores de Risco e Proteção para Doenças Crônicas em Inquérito Telefônico.

[29] Instituto Brasileiro de Geografia e Estatística (2010). Censo Demográfico 2010: Resultados gerais da população, domicílios e características sociodemográficas.

